# Amino acid deprivation promotes intestinal homeostasis through autophagy

**DOI:** 10.18632/oncotarget.8841

**Published:** 2016-04-19

**Authors:** Lorenzo Galluzzi, Guido Kroemer

**Affiliations:** Equipe 11 labellisée Ligue contre le Cancer, Centre de Recherche des Cordeliers, Paris, France; INSERM, U1138, Paris, France; Université Paris Descartes/Paris V, Sorbonne Paris Cité, Paris, France; Université Pierre et Marie Curie/Paris VI, Paris, France; Gustave Roussy Comprehensive Cancer Institute, Villejuif, France; Equipe 11 labellisée Ligue contre le Cancer, Centre de Recherche des Cordeliers, Paris, France; INSERM, U1138, Paris, France; Université Paris Descartes/Paris V, Sorbonne Paris Cité, Paris, France; Université Pierre et Marie Curie/Paris VI, Paris, France; Metabolomics and Cell Biology Platforms, Gustave Roussy Comprehensive Cancer Institute, Villejuif, France; Pôle de Biologie, Hôpital Européen Georges Pompidou, AP-HP, Paris, France; Department of Women's and Children's Health, Karolinska University Hospital, Stockholm, Sweden

**Keywords:** eIF2α, GCN2, inflammasome, mitophagy, reactive oxygen species

Macroautophagy (here below referred to as autophagy) is a fundamental gatekeeper of intracellular homeostasis. On the one hand, autophagy operates at baseline levels to dispose of damaged organelles and other potentially cytotoxic byproducts of normal cellular functions, hence preserving metabolic and redox fitness in physiological conditions [1]. On the other hand, the autophagic machinery is functionally connected to sensors that continuously monitor the intracellular and extracellular milieu for chemical, physical, biological, infectious and metabolic threats. Thus, cells continuously adapt autophagic flux (i.e., the actual degradation of autophagic substrates by lysosomes) to cope with potentially dangerous fluctuations of homeostasis [2]. Corroborating the central cytoprotective function of autophagy, pharmacological agents or genetic interventions that inhibit one fundamental component of the autophagic machinery most often precipitate the demise of cells responding to stress [3]. Accumulating data indicate that autophagy does not only occupy a key position in cell-intrinsic responses to stress, but also stands at the hub of several cell-extrinsic mechanisms of preservation of organismal homeostasis [4]. Ravindran *et al.* recently demonstrated that the amino acid-responsive sensor eukaryotic translation initiation factor 2 alpha kinase 4 (EIF2AK4; best known as general control nonderepressible 2, GCN2) dampens intestinal inflammation *via* a cell-extrinsic mechanism that involves autophagy [5]. These findings lend further support to the conjecture that the capacity of autophagy to preserve homeostasis trespasses the virtual boundary represented by the plasma membrane.

Ravindran and colleagues observed that GCN2, as well as other kinases that phosphorylate eukaryotic translation initiation factor 2A (EIF2A, best known as eIF2α), are overexpressed and activated in the gut of patients with ulcerative colitis or Crohn's disease. They therefore set out to investigate the intestinal phenotype of *Eif2ak4^−/−^* mice, finding that GCN2 is not required for gut homeostasis in baseline conditions. However, *Eif2ak4^−/−^* mice were more sensitive to dextran sodium sulfate (DSS)-driven colitis than their wild-type (WT) littermates, as they exhibited comparatively more severe weight loss, local inflammation with a prominence of T_H_17 cells, and colon shortening. Such an inflammatory phenotype could be attributed to the loss of GCN2 in antigen-presenting cells (APCs) and intestinal epithelial cells (IECs), and was specific to GCN2, as mice lacking the eIF2α kinase PERK in APCs or IECs were as sensitive to DSS-induced colitis as WT animals. Moreover, mice in which a non-phosphorylatable variant of eIF2α (eIF2α^S51A^) was conditionally expressed in APCs or epithelial cells displayed a less severe pathological phenotype than *Eif2ak4^−/−^* mice, demonstrating that GCN2 limits the susceptibility of mice to DSS-driven inflammation *via* eIF2α-dependent and eIF2α-independent mechanisms [5].

Since GCN2 and other eIF2α kinases also promote autophagy [6], Ravindran and colleagues decided to cross *Eif2ak4^−/−^* mice with mice expressing a fluorescent reporter of autophagy, observing that DSS administration promoted robust autophagic responses in APCs and IECs only when GCN2 was expressed. Mice lacking either of two essential components of the autophagic machinery - that is, autophagy-related 5 (ATG5) or ATG7 - in APCs also exhibited increased weight loss, inflammation and colon shortening upon administration of DSS, demonstrating that defective autophagy has indeed an etiological role in this setting. Such a pathological effect was linked to the ability of proficient autophagic responses to preserve mitochondrial fitness and limit the accumulation of reactive oxygen species (ROS) in APCs [7], hence avoiding the activation of the inflammasome, the release of mature interleukin-1β (IL-1β) and IL-18, and the consequent initiation of an inflammatory T_H_17 response. Importantly, WT (but not *Eif2ak4^−/−^*) mice maintained on a standard diet (16% protein) were more sensitive to a severe DSS challenge than mice on a low-protein diet (2% protein) or on a diet specifically lacking leucine (16% protein) [5].

Taken together, the findings by Ravindran and colleagues demonstrate that autophagic responses driven by nutritional cues (in this case, amino acid availability) can contribute to the preservation of homeostasis not only at the cell-intrinsic, but also at the cell-extrinsic level. This has far-reaching implications for the management of multiple diseases in which inter-cellular communication is altered, including (but not limited to) inflammatory conditions.
